# An Effective Approach for NRSFM of Small-Size Image Sequences

**DOI:** 10.1371/journal.pone.0132370

**Published:** 2015-07-10

**Authors:** Ya-Ping Wang, Zhan-Li Sun, Kin-Man Lam

**Affiliations:** 1 School of Electrical Engineering and Automation, Anhui University, Hefei, China; 2 Department of Electronic and Information Engineering, Hong Kong Polytechnic University, Hong Kong, China; Universitat Rovira i Virgili, SPAIN

## Abstract

In recent years, non-rigid structure from motion (NRSFM) has become one of the hottest issues in computer vision due to its wide applications. In practice, the number of available high-quality images may be limited in many cases. Under such a condition, the performances may not be satisfactory when existing NRSFM algorithms are applied directly to estimate the 3D coordinates of a small-size image sequence. In this paper, a sub-sequence-based integrated algorithm is proposed to deal with the NRSFM problem with small sequence sizes. In the proposed method, sub-sequences are first extracted from the original sequence. In order to obtain diversified estimations, multiple weaker estimators are constructed by applying the extracted sub-sequences to a recent NRSFM algorithm with a rotation-invariant kernel (RIK). Compared to other first-order statistics, the trimmed mean is a relatively robust statistic. Considering the fact that the estimations of some weaker estimators may have large errors, the trimmed means of the outputs for all the weaker estimators are computed to determine the final estimated 3D shapes. Compared to some existing methods, the proposed algorithm can achieve a higher estimation accuracy, and has better robustness. Experimental results on several widely used image sequences demonstrate the effectiveness and feasibility of the proposed algorithm.

## Introduction

Non-rigid structure from motion (NRSFM) is the process of recovering the relative camera motion, and the time-varying 3D coordinates of feature points on a deforming object, by means of the corresponding 2D points in a sequence of images. In many cases, the recovered 3D shapes can effectively enhance the performances of existing systems in object recognition, face perception, etc. [1–3]. Nevertheless, in the NRSFM model, the objects generally undergo a series of shape deformations and pose variations. Thus, in the absence of necessary prior knowledge on shape deformation, recovering the 3D shape and motion of nonrigid objects from 2D point tracks remains a difficult and ill-posed problem.

As a pioneering work, a non-rigid model was proposed in [4] by formulating the 3D shape in each frame of a sequence as a linear combination of a set of basis shapes. Nevertheless, due to a lack of sufficient constraints on the shape deformation, the recovered 3D shapes are not unique under this model. In order to alleviate the ambiguities, recent research works have attempted to define additional constraints to make NRSFM more tractable [5]. More determined solutions are given in [6] by utilizing the facts that the bases degenerate under some special cases. In [7, 8], the 3D shape at each time instant is assumed to be drawn from a Gaussian distribution. Assuming that the 3D shape deformation is smooth over time, the time-varying structure of a nonrigid object is represented as a linear combination of a set of basis trajectories [9–11], e.g. the Discrete Cosine Transform (DCT) basis. Since the basis trajectories are known *a priori*, this method can significantly reduce the number of unknown parameters and improve the estimation stability. Instead of the time-varying structure, the camera’s trajectory is modeled as a linear combination of DCT basis vectors, which provides better results on complex articulated deformations [12, 13]. In [14], the complex deformable 3D shapes are represented as the outputs of a non-linear mapping via the kernel trick [15]. Recently, a novel NRSFM with a rotation-invariant kernel (RIK) was proposed in [16], which utilizes the spatial-variation constraint. A prominent advantage of this method is that it is able to deal with the data lacking temporal ordering or with abrupt deformations.

In practice, the number of available high-quality images may be limited in many cases, such as the face images in a surveillance system, etc. If the existing NRSFM algorithms are directly used to estimate the 3D coordinates of a small-size image sequence, the estimation accuracy may be relatively low. In this paper, a sub-sequence based integrated algorithm is proposed to deal with the small-sequence problem. In the proposed method, the 3D coordinates of each frame are estimated one by one. For a test frame, except for itself, a few frames are first randomly extracted from the original sequence. Then, the extracted frames, together with the test frame, form a sub-sequence to be applied to RIK. Similar to the classifier committee learning [17], the sub-sequence and the estimation process of RIK constitute a weaker estimator. Finally, the *z*-coordinates obtained by multiple weaker estimators are integrated and used as the final estimation for the test frame. Experimental results on several widely used image sequences demonstrate the effectiveness and feasibility of the proposed algorithm.

## Methodology

**Fig 1:**
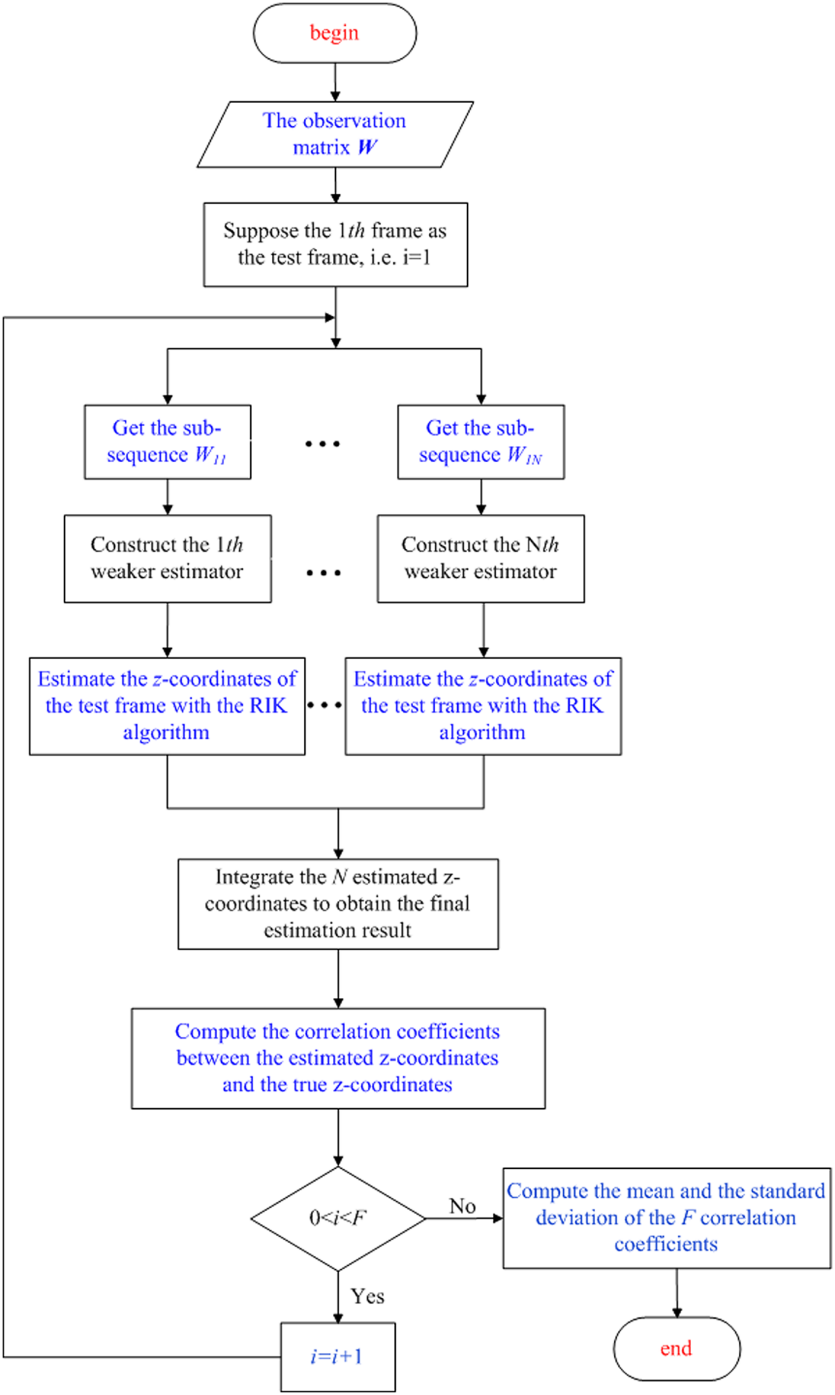
Flowchart of the sub-sequence-based integrated RIK algorithm.

Fig 1 shows the flowchart of the sub-sequence-based integrated RIK algorithm. There are three main steps in our algorithm: extract the sub-sequences from the original sequences, construct the weaker estimators based on the RIK algorithm, and integrate the outputs of the weaker estimators. A detailed description of these three steps is presented in the following subsections.

### Sub-Sequence Extraction

**Fig 2:**
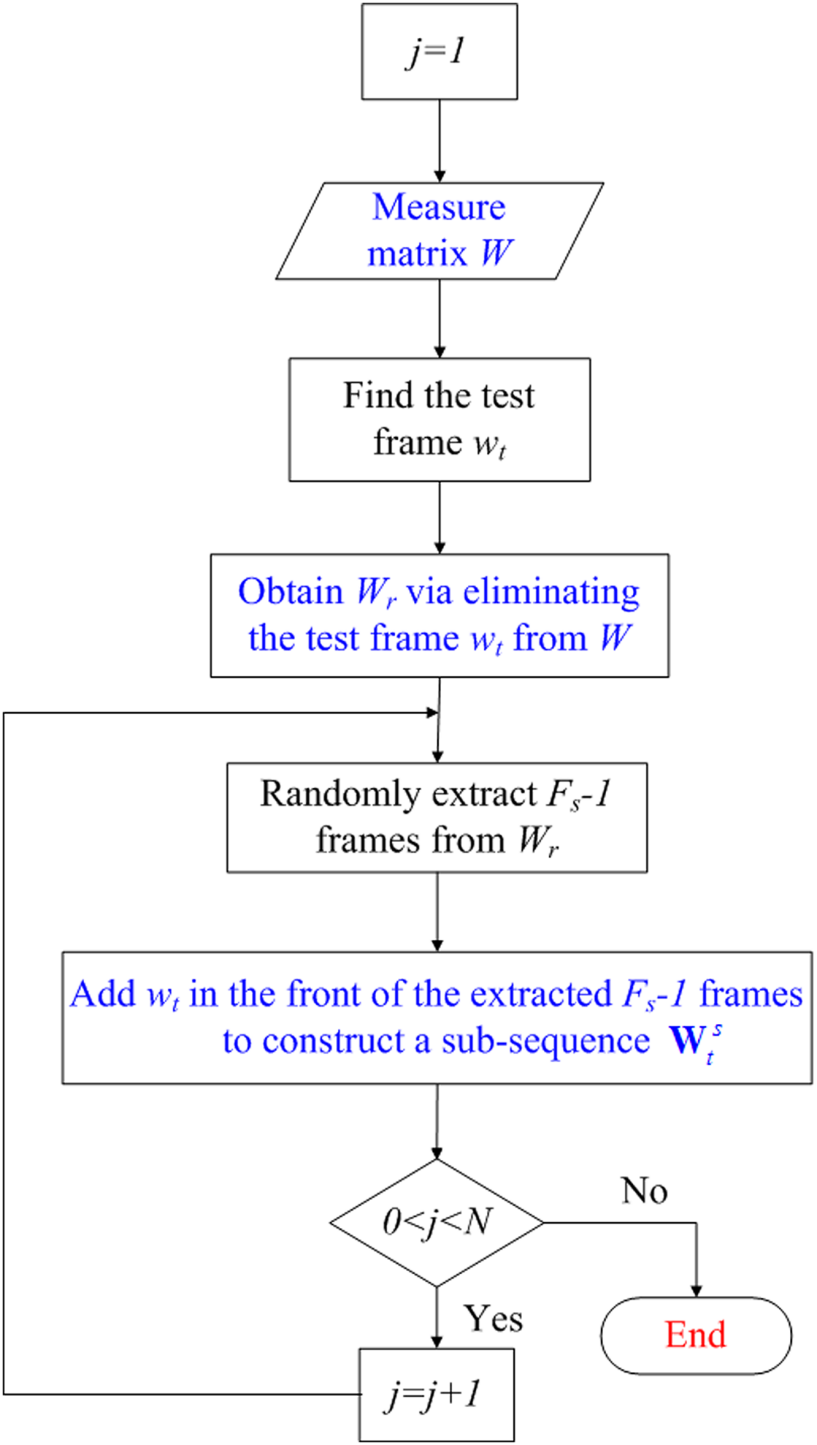
The extraction of sub-sequences.

The first step of our proposed method is to extract sub-sequences from a small-size sequence, as shown in Fig 2. For a sequence with *F* frames and *n* feature points in each of the frames, denote [*x*_*t*, *j*_, *y*_*t*, *j*_]^*T*^ (*t* = 1, 2, ⋯, *F*, *j* = 1, 2, ⋯, *n*) as the 2D projection of the *j*th 3D point observed on the *t*th image. The *n* 2D point tracks of the *F* images can be represented as a 2*F* × *n* observation matrix **W**, i.e.

$$\begin{array}{c} W=(\begin{array}{cccc} x_{1,1} & x_{1,2} & \cdots & x_{1,n}\\ y_{1,1} & y_{1,2} & \cdots & y_{1,n}\\ \vdots & \vdots & \ddots & \vdots \\ x_{F,1} & x_{F,2} & \cdots & x_{F,n}\\ y_{F,1} & y_{F,2} & \cdots & y_{F,n} \end{array}). \end{array}$$ (1)

For the *t*th frame, the observation **w**_*t*_ is a 2 × *n* matrix, as follows:

$$\begin{array}{c} w_{t}=(\begin{array}{cccc} x_{t,1} & x_{t,2} & \cdots & x_{1,n}\\ y_{t,1} & y_{t,2} & \cdots & y_{1,n} \end{array}). \end{array}$$ (2)

The observations of an original sequence with *F* images are derived. When the 3D coordinates of the *t*th image are to be estimated, the matrix **W**_*r*_ shown in Fig 2 can be given as follows:

$$W_{r}={\left[w_{1}^{T},\cdots ,w_{t-1}^{T},w_{t+1}^{T},\cdots ,w_{F}^{T}\right]}^{T}.$$ (3)

Assuming that the number of frames in a sub-sequence is *F*_*s*_, the observation matrix ${\text{W}}_{t}^{s}$ is constructed by randomly selecting *F*_*s*_−1 observations from **W**_*r*_ and merging them with **w**_*t*_. Thus, *N* sub-sequences ${\text{W}}_{tj}^{s}(j=1,\cdots ,N)$ are obtained when the sub-sequence extraction process is repeated *N* times.

### RIK-based Weaker Estimator

For each test frame **w**_*t*_, we construct *N* sub-sequence observation matrices ${\text{W}}_{tj}^{s}(j=1,\cdots ,N)$. In order to estimate the 3D coordinates of the *t*th frame, one sub-sequence ${\text{W}}_{tj}^{s}$ is applied to the RIK algorithm. Assume that the number of basis shapes is *K*. In terms of the linear-subspace model [8], ${\text{W}}_{tj}^{s}$ is factorized as a product of two matrices via singular value decomposition, i.e.

$$W_{tj}^{s}=MS,$$ (4)

where **M** is a 2*F*_*s*_ × 3*K* camera matrix, and **S** includes *K* basis shapes, i.e.

$$S=[\begin{array}{c} {\hat{S}}_{1}\\ \vdots \\ {\hat{S}}_{K} \end{array}]\in {\mathbb{R}}^{3K\times n}.$$ (5)

Further, **M** is decomposed as follows:

$$M=D(C⊗I_{3}),$$ (6)

where the block-diagonal rotation matrix **D** is obtained via an Euclidean upgrade step [10], and **C** and **I**_3_ represent a shape coefficient matrix and a 3 × 3 identity matrix, respectively. The operator ⊗ denotes the Kronecker product. Further, **C** is represented as a product of the coefficient matrix **X** and a new basis matrix **B** [13], i.e.

C=BX. (7)

In the optimization procedure, **X** can be initialized as a low-rank identity matrix, and **B** is computed via the kernel mapping [15]. Let ${\text{c}}_{t}^{T}$ be the *t*th row of **C**. The 3D shape of the *t*th image can be given as follows:

$$S\left(c_{t}^{T}\right)=\left(c_{t}^{T}⊗I_{3}\right)M^{†}W_{tj}^{s},$$ (8)

where **M** ^†^ denotes the Moore-Penrose pseudo-inverse of **M** [16].

### Integration of Weaker Estimators

For the *t*th test frame, we can see from Section 1 that one set of estimated **z**_*tj*_ can be obtained for the *j*th sub-sequence ${\text{W}}_{tj}^{s}$. When each sub-sequence ${\text{W}}_{tj}^{s}(j=1,\cdots ,N)$ is applied in turn to RIK, we can obtain *N* sets of estimated **z**_*tj*_ (*j* = 1, ⋯, *N*). Similar to the notation of classifier-committee learning [17] in pattern recognition, here each input ${\text{W}}_{tj}^{s}$ and the corresponding reconstruction model can be considered as a weaker estimator. In order to integrate the results obtained by the *N* weaker estimators, the arithmetic average ${\hat{\text{z}}}_{t}$ of **z**_*t*1_, ⋯, **z**_*tN*_ is a relatively simple implementation, i.e.

$${\hat{z}}_{t}=\frac{1}{N}\sum_{j=1}^{N}z_{tj},$$ (9)

which can be used as the final estimated z-coordinates of the *t*th test image. Compared to the arithmetic average, the trimmed mean is a more robust integration estimation. Assuming that *P* percentage of the observations is trimmed, the number (*N*_*d*_) of the smallest or the largest observations to be discarded is

$$N_{d}=\left[N\left(P/100\right)/2\right],$$ (10)

where [⋅] denotes a rounding operation. Further, assuming that the entries of **z**_*tj*_ are ordered such that **z**_*t*1_ < **z**_*t*2_ < ⋯ < **z**_*tN*_, the trimmed mean ${\hat{\text{z}}}_{t}$ can be computed as follows:

$${\hat{z}}_{t}=\frac{1}{N-2N_{d}}\sum_{j=N_{d}+1}^{N-N_{d}}z_{tj},\;t=1,\cdots ,F.$$ (11)

## Experimental results

### Experimental data

We evaluate the performance of our proposed method on three synthetic-image sequences (*stretch*, *face1*, *face2*) and three real-image sequences (*cubes, dance, matrix*), which are widely used sequences and are publicly available [11, 16]. For these 6 sequences, the corresponding number of frames (*T*) and the number of point tracks (*n*) are shown in Table 1.

**Table 1:** The number of frames (*T*) and the number of point tracks (*n*) for 6 sequences.

	Sequences	*T*	*n*
1	stretch	370	41
2	face1	74	37
3	face2	316	40
4	cubes	200	14
5	matrix	105	30
6	dance	264	75

Besides these data, some real face-image sequences from the Bosphorus database are also used in the experiments. Bosphorus is a relatively new 3D face database that includes face images with a rich set of expressions and a systematic variation in poses [18].

To evaluate the estimation accuracy, two performance indices are adopted here to compare the true 3D shapes and the estimated results. One performance index is the Pearson’s linear correlation coefficient $c(\text{z},\hat{\text{z}})$ between the true z-coordinates **z** and the estimated z-coordinates $\hat{\text{z}}$, i.e.

$$c\left(z,\hat{z}\right)=\frac{1}{n-1}\sum_{i=1}^{n}\left(\frac{z_{i}-\mu_{z}}{\sigma_{z}}\right)\left(\frac{{\hat{z}}_{i}-\mu_{\hat{z}}}{\sigma_{\hat{z}}}\right),$$ (12)

where *μ*_*z*_ and *σ*_*z*_ are the respective mean and standard deviation of **z**, and $\mu_{\hat{z}}$ and $\sigma_{\hat{z}}$ are the respective mean and standard deviation of $\hat{\text{z}}$. A higher absolute value of $c(\text{z},\hat{\text{z}})$ means that $\hat{\text{z}}$ is closer to **z**. The other performance index is the mean error $\epsilon (\text{z},\hat{\text{z}})$ between the true z-coordinates **z** and the estimated z-coordinates $\hat{\text{z}}$, i.e.

$$\epsilon \left(z,\hat{z}\right)=\frac{1}{n}\sum_{i=1}^{n}\left|\left(z_{i}-{\hat{z}}_{i}\right)\right|,$$ (13)

### Experiments

In order to verify the performance of our proposed sub-sequence-based integrated RIK algorithm (denoted as SSI-RIK), we compare it to the original RIK method [16], EM-SFM [7], and CSF [14], which have relatively good performances among existing algorithms.

As the challenge addressed in this paper is the NRSFM problem with small-size image sequences, we first extract a small sequence from an original sequence, to be used as the experimental data. Take the sequence *stretch*, for example: the first 15 frames are used to form a small sequence. i.e. *F* = 15. The length of sub-sequences (*F*_*s*_) and the number of weaker estimators (*N*) are set at 6 and 10, respectively. For the four algorithms, Table 2 shows the correlation coefficients of the 15 frames, and the corresponding mean (*μ*) and standard deviation (*σ*). Table 3 shows the correlation coefficient increasing percentages (%) of SSI-RIK compared to EM-SFM, CSF and RIK. Additionally, Tables 4 and 5 show the similar performance comparisons of the *z*-coordinate errors. In these Tables, the numbers 1 to 15 denote the 1th to 15th frame in the small sequence.

**Table 2:** The correlation coefficients, and the corresponding mean (*μ*) and standard deviation (*σ*), of 15 frames of the sequence *stretch* for 4 algorithms.

Frame Number	EM-SFM	CSF	RIK	SSI-RIK
1	0.1873	0.6087	0.1700	0.9813
2	0.1997	0.6459	0.2425	0.9879
3	0.2083	0.6966	0.3295	0.9859
4	0.2134	0.7719	0.4602	0.9882
5	0.1997	0.8420	0.6325	0.9944
6	0.1632	0.9011	0.8696	0.9915
7	0.1426	0.9612	0.9205	0.9954
8	0.1138	0.9921	0.9497	0.9960
9	0.1007	0.9893	0.9601	0.9946
10	0.0728	0.9554	0.9551	0.9934
11	0.0375	0.8951	0.9207	0.9903
12	0.0268	0.8120	0.9010	0.9874
13	0.0219	0.7251	0.8933	0.9802
14	0.0224	0.6436	0.8843	0.9817
15	0.0262	0.5705	0.8711	0.9762
*μ*	0.1157	0.8007	0.7307	0.9883
*σ*	0.0765	0.1461	0.2851	0.0062

**Table 3:** The correlation coefficient increasing percentages (%) of SSI-RIK compared to EM-SFM, CSF and RIK.

Frame Number	$(\frac{SSI-RIK}{EM-SFM}-1)*100$	$(\frac{SSI-RIK}{CSF}-1)*100$	$(\frac{SSI-RIK}{RIK}-1)*100$
1	423.9241	61.2107	477.2671
2	394.6490	52.9373	307.3797
3	373.4054	41.5431	199.2156
4	362.9820	28.0223	114.7493
5	398.0821	18.1051	57.2236
6	507.6249	10.0365	14.0165
7	597.9663	3.5580	8.1411
8	775.5285	0.3979	4.8737
9	888.1652	0.5341	3.5889
10	1264.5	3.9763	4.0102
11	2540.4	10.6278	7.5507
12	3588.5	21.5968	9.5802
13	4366.2	35.1860	9.7305
14	4284.4	52.5452	11.0215
15	3619.7	71.1259	12.0690
*μ*	753.8268	23.4300	35.2578

**Table 4:** The *z*-coordinate errors, and the corresponding mean (*μ*) and standard deviation (*σ*), of 15 frames of the sequence *stretch* for 4 algorithms.

Frame Number	EM-SFM	CSF	RIK	SSI-RIK
1	0.5783	0.2936	0.4793	0.0420
2	0.5353	0.2680	0.4400	0.0370
3	0.4960	0.2350	0.3868	0.0441
4	0.4602	0.1852	0.3067	0.0388
5	0.4247	0.1433	0.2165	0.0309
6	0.4007	0.1078	0.1109	0.0295
7	0.3989	0.0630	0.0833	0.0218
8	0.3948	0.0269	0.0720	0.0210
9	0.3934	0.0304	0.0645	0.0273
10	0.3946	0.0661	0.0640	0.0284
11	0.3997	0.1066	0.0922	0.0325
12	0.4033	0.1514	0.1082	0.0357
13	0.4068	0.1963	0.1130	0.0449
14	0.4110	0.2403	0.1169	0.0422
15	0.4118	0.2843	0.1227	0.0497
*μ*	0.4342	0.1599	0.1851	0.0351
*σ*	0.0577	0.0917	0.1439	0.0086

**Table 5:** The *z*-coordinate error decreasing percentages (%) of SSI-RIK compared to EM-SFM, CSF and RIK.

Frame Number	$(1-\frac{SSI-RIK}{EM-SFM})*100$	$(1-\frac{SSI-RIK}{CSF})*100$	$(1-\frac{SSI-RIK}{RIK})*100$
1	92.7335	85.6856	91.1333
2	93.0961	86.2091	91.6044
3	91.1128	81.2403	88.6056
4	91.5725	79.0604	87.3552
5	92.7245	78.4350	87.7288
6	92.6255	72.5794	73.3644
7	94.5323	65.3521	75.2942
8	94.6739	21.8112	70.8067
9	93.0505	10.1219	57.6105
10	92.8134	57.0992	55.6592
11	91.8814	69.5715	64.8051
12	91.1454	76.4122	66.9998
13	88.9547	77.1146	60.2482
14	89.7311	82.4328	63.9039
15	88.0274	82.5346	59.5209
*μ*	91.9268	78.0756	81.0650

From Tables 2 and 3, we can see that the correlation coefficients of SSI-RIK are obviously higher than those of EM-SFM, CSF and RIK. Moreover, it can be seen from Tables 4 and 5 that the *z*-coordinate errors of SSI-RIK are significantly lower than those of EM-SFM, CSF and RIK. Thus, SSI-RIK has a higher estimation accuracy than the other methods. In addition, we can see from Tables 2 and 4 that the standard deviations of SSI-RIK are lower than those of the other three methods. This indicates that SSI-RIK is a more robust approach.

**Fig 3:**
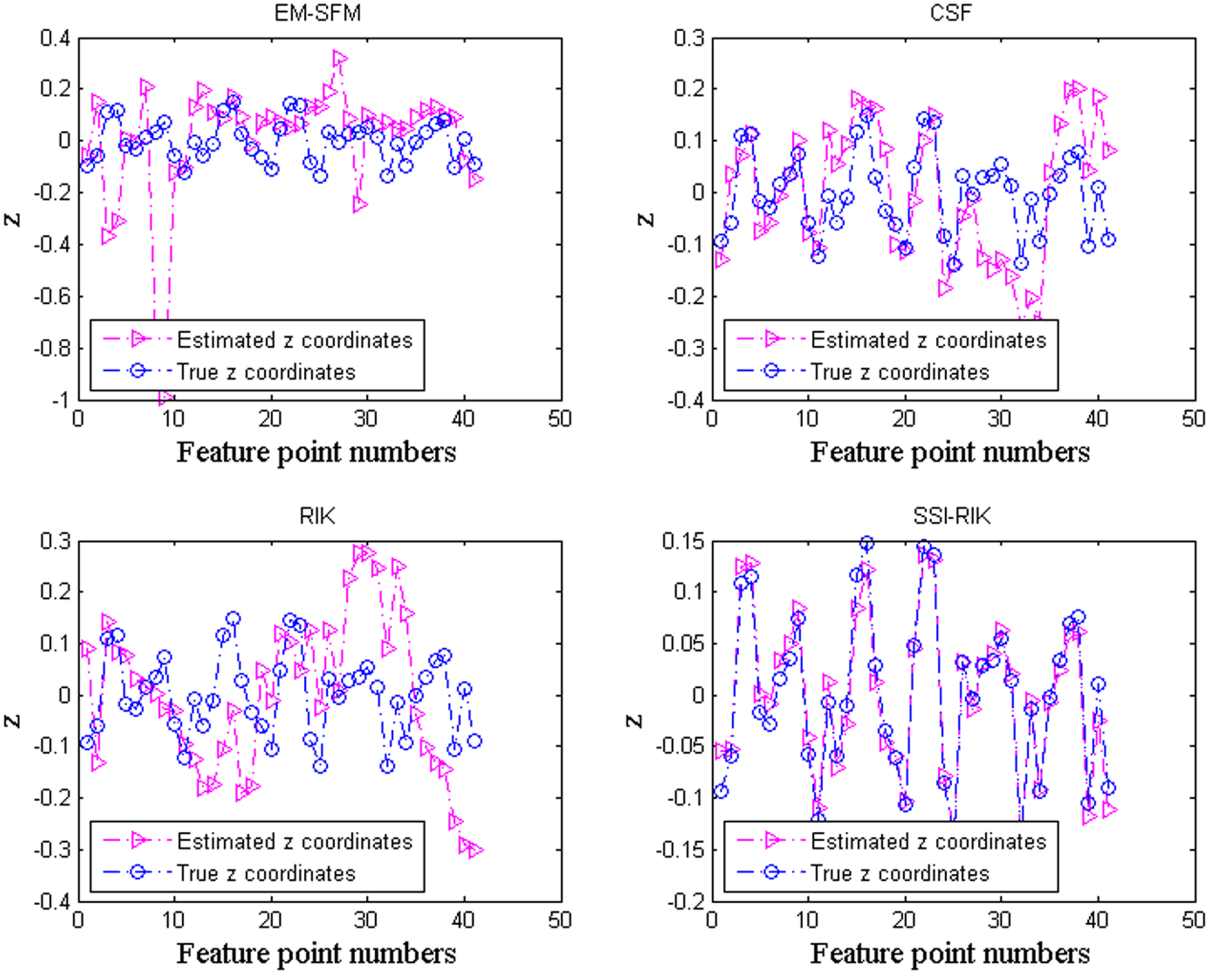
The comparisons of the true z-coordinate values and the estimated z-coordinate values of the first frame of *stretch* for the four methods.

**Fig 4:**
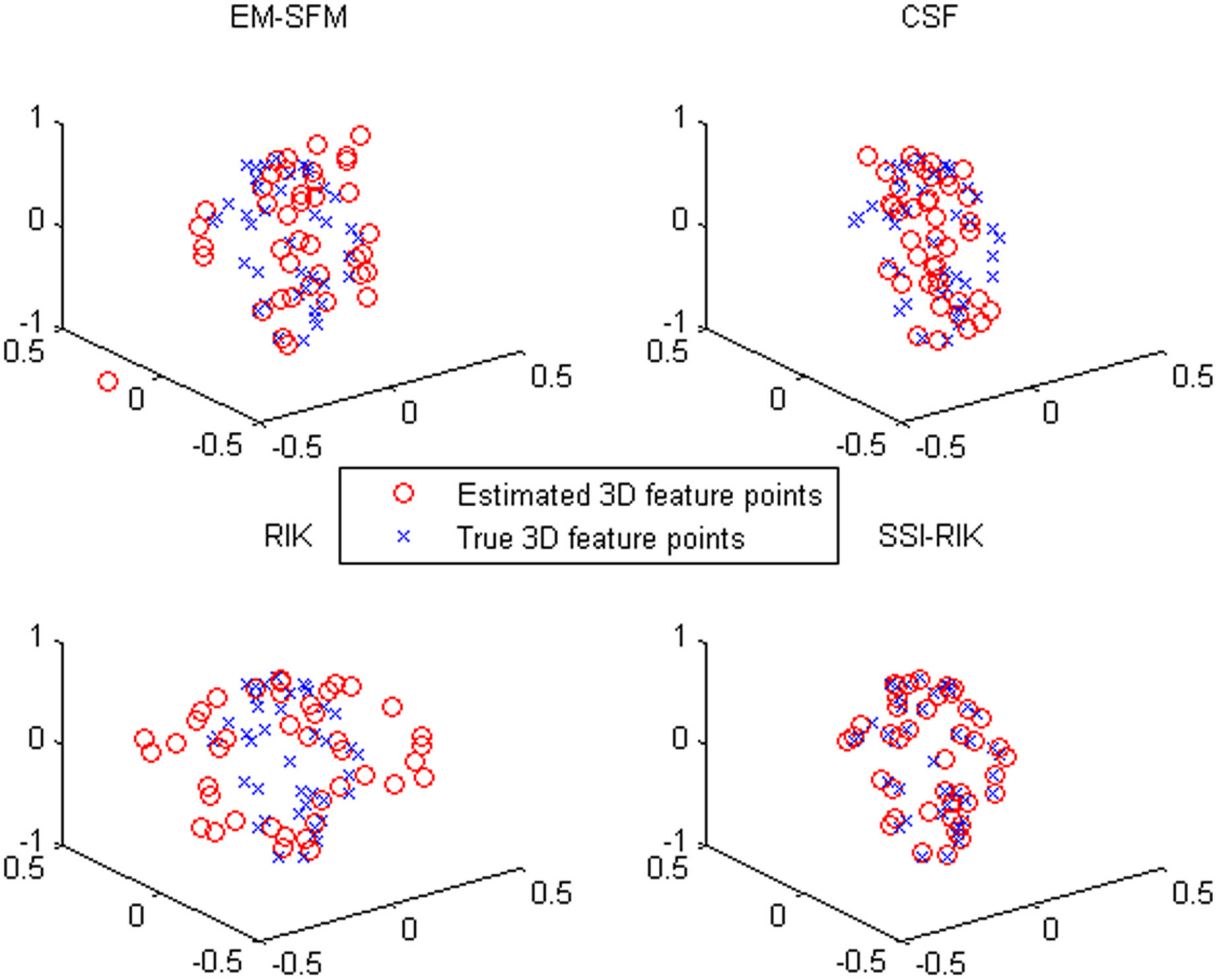
The comparisons of the true 3D feature points and the estimated 3D feature points of the first frame of *stretch* for the four methods.

Taking the first frame of *stretch* as an example, Figs 3 and 4 show the comparisons of the true values and the estimated values for the *z*-coordinate values and the 3D feature points, respectively. We can see that the *z*-coordinate values and the 3D feature points estimated by SSI-RIK are closer to the true values than those estimated by the other three methods, which coincides with the performance indices of the correlation coefficients and the *z*-coordinate errors.

In order to investigate the effect of sequence size (*F*) on the performances of the various algorithms, Tables 6 and 7 tabulate the mean and standard deviation (*μ* ± *σ*) of the correlation coefficients and the *z*-coordinates errors, respectively, when the sequence sizes vary from 15 to 50 with an equal interval of 5. Moreover, for the mean values of the correlation coefficients and the *z*-coordinates errors, Tables 8 and 9 show the corresponding increasing percentages and decreasing percentages of SSI-RIK compared to EM-SFM, CSF and RIK, respectively.

**Table 6:** The mean and standard deviation (*μ* ± *σ*) of correlation coefficients when the sequence sizes vary from 15 to 50 with an equal interval 5.

	EM-SFM	CSF	RIK	SSI-RIK
15	0.1157±0.0765	0.8007±0.1461	0.7307±0.2851	0.9883±0.0062
20	0.3508±0.0568	0.7093±0.1841	0.9702±0.0204	0.9868±0.0114
25	0.3104±0.0998	0.6626±0.2156	0.9659±0.0318	0.9870±0.0134
30	0.3042± 0.1175	0.5896±0.2455	0.8659±0.1565	0.9763±0.0213
35	0.1448± 0.0897	0.5185±0.2706	0.9705±0.0449	0.9708±0.0220
40	0.3888±0.0767	0.4432±0.2983	0.9490±0.0527	0.9719±0.0191
45	0.4564±0.0666	0.3875±0.3108	0.8095±0.2874	0.9678±0.0393
50	0.1167±0.0752	0.3658±0.3024	0.8296±0.2427	0.9710±0.0330

**Table 7:** The mean and standard deviation (*μ* ± *σ*) of *z*-coordinate errors when the sequence sizes vary from 15 to 50 with an equal interval 5.

	EM-SFM	CSF	RIK	SSI-RIK
15	0.4342±0.0245	0.1599±0.0917	0.1851±0.1439	0.0351±0.0086
20	1.0501±0.0064	0.2217±0.1196	0.0551±0.0177	0.0352±0.0148
25	1.6247±0.0107	0.2620±0.1441	0.0522±0.0238	0.0328±0.0176
30	1.5909±0.0137	0.3101±0.1635	0.1028±0.0549	0.0409±0.0198
35	1.5841±0.0140	0.3534±0.1751	0.0488±0.0259	0.0473±0.0210
40	1.7107±0.0059	0.3935±0.1840	0.0666±0.0365	0.0482±0.0175
45	1.1868±0.0115	0.4153±0.1835	0.1504±0.1529	0.0478±0.0279
50	0.4857 ±0.0125	0.4232±0.1701	0.1341±0.1255	0.0458±0.0214

**Table 8:** The mean correlation coefficient increasing percentages (%) of SSI-RIK compared to EM-SFM, CSF and RIK, when the sequence sizes vary from 15 to 50 with an equal interval 5.

	$(\frac{SSI-RIK}{EM-SFM}-1)*100$	$(\frac{SSI-RIK}{CSF}-1)*100$	$(\frac{SSI-RIK}{RIK}-1)*100$
15	753.8268	23.4300	35.2578
20	181.2612	39.1249	1.7095
25	217.9668	48.9574	2.1781
30	220.9478	65.6084	12.7546
35	570.2348	87.2287	0.0319
40	149.9892	119.3056	2.4215
45	112.0418	149.7742	19.5537
50	732.0983	165.4305	17.0438

**Table 9:** The mean *z*-coordinate error decreasing percentages (%) of SSI-RIK compared to EM-SFM, CSF and RIK, when the sequence sizes vary from 15 to 50 with an equal interval 5.

	$(1-\frac{SSI-RIK}{EM-SFM})*100$	$(1-\frac{SSI-RIK}{CSF})*100$	$(1-\frac{SSI-RIK}{RIK})*100$
15	91.9268	78.0756	81.0650
20	96.6438	84.1055	36.0142
25	97.9831	87.4934	37.1732
30	97.4313	86.8213	60.2506
35	97.0155	86.6232	3.0704
40	97.1840	87.7593	27.6980
45	95.9684	88.4783	68.1849
50	90.5625	89.1686	65.8147

**Fig 5:**
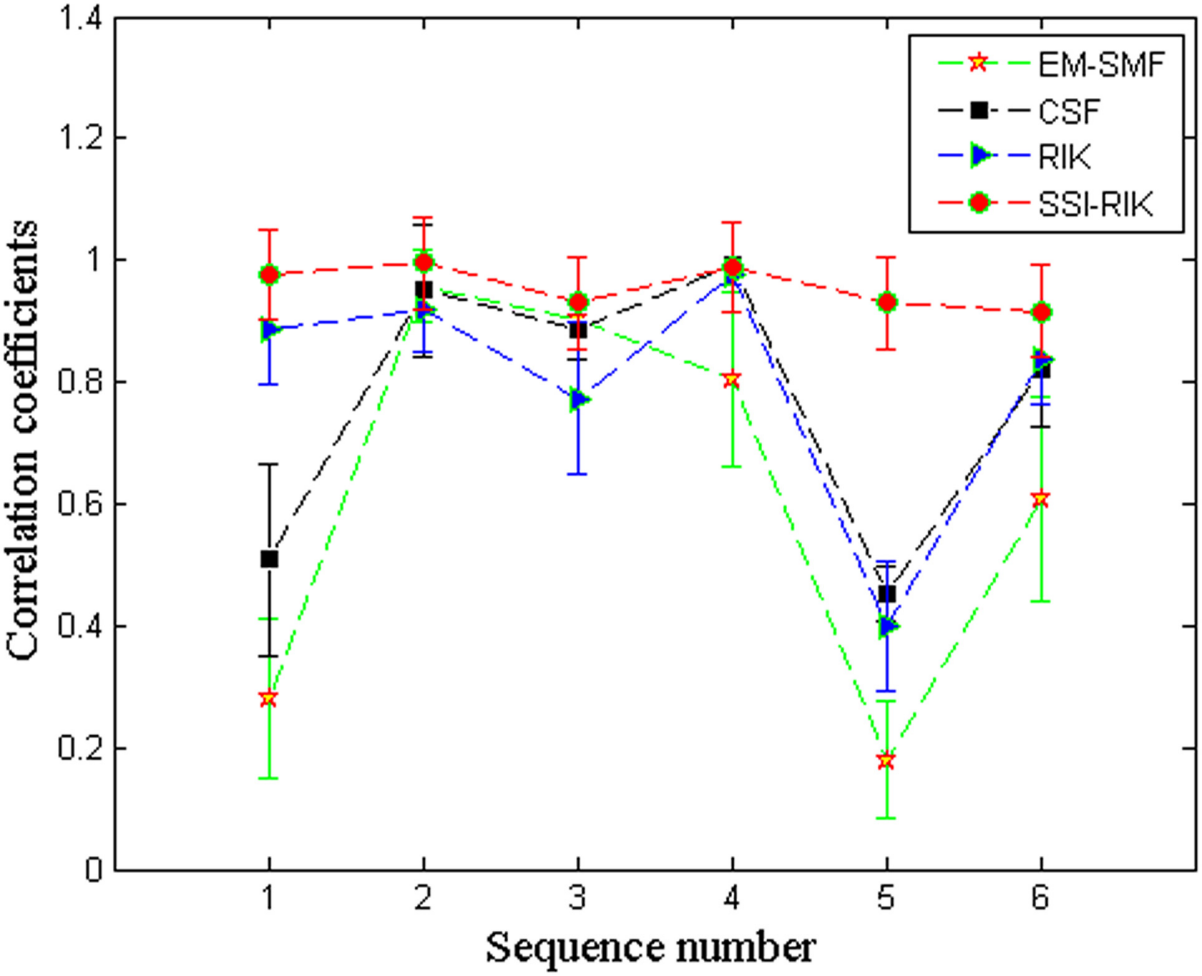
The overall mean and standard deviation (*μ* ± *σ*) of correlation coefficients for different sequence sizes.

**Fig 6:**
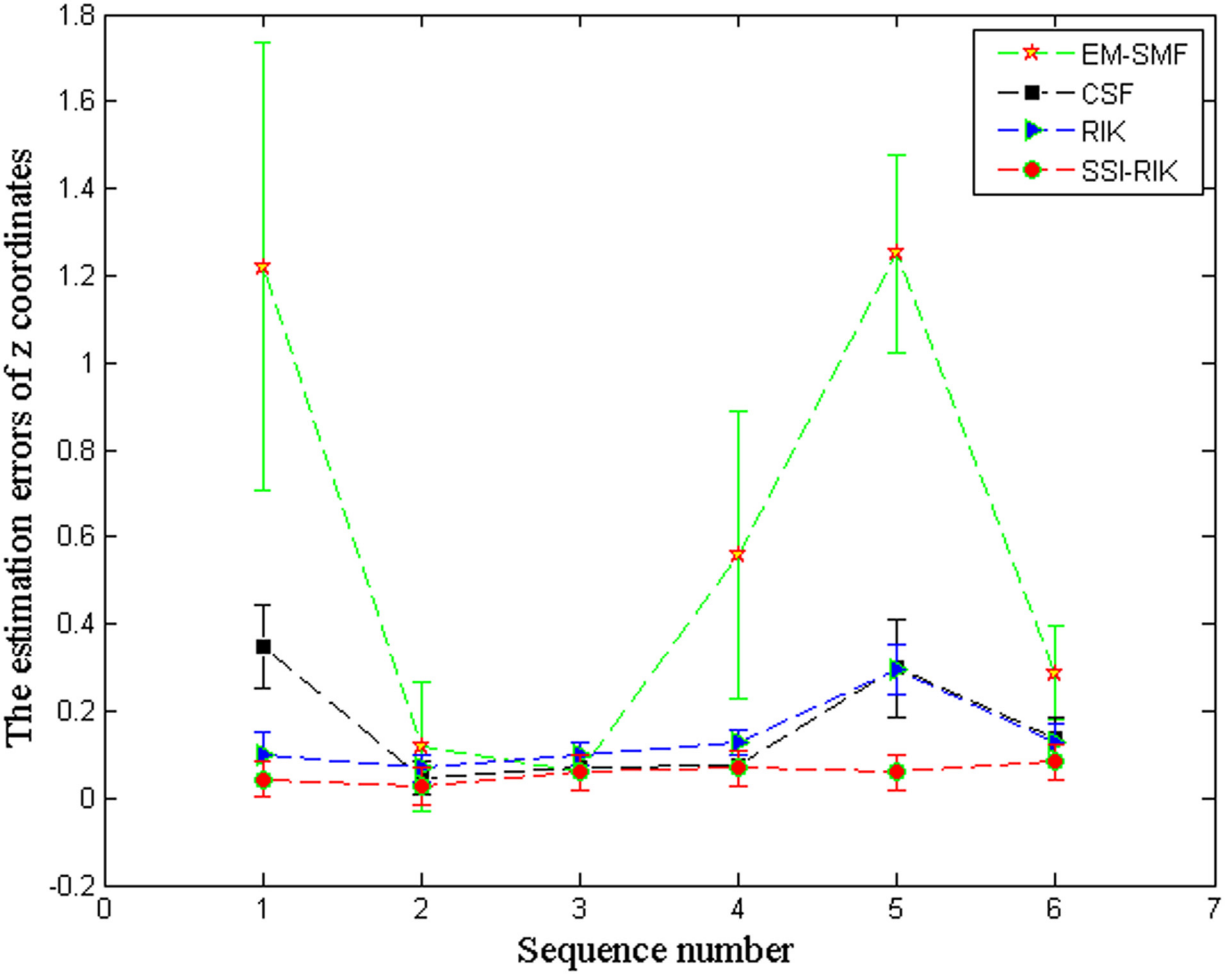
The overall mean and standard deviation (*μ* ± *σ*) of *z*-coordinate errors for different sequence sizes.

Further, Figs 5 and 6 show the overall mean and standard deviation (*μ* ± *σ*) of the correlation coefficients and the *z*-coordinate errors for different sequence sizes, respectively. In these two figures, the *x* axis denotes image sequences in terms of the numbers shown in Table 1. From Tables 6–9 and Figs 5 and 6, we can see that SSI-RIK has a better performance than EM-SFM, CSF and RIK for different sequence sizes.

We also present the experimental results on the real Bosphorus database. In experiments, the *z*-coordinates of the frontal-view images are estimated. As an example, Tables 10 and 11 show the correlation coefficients and the *z*-coordinate errors, respectively, when the sequence sizes vary from 7 to 14 for one individual. Moreover, Tables 12 and 13 show the corresponding increasing and decreasing percentages of SSI-RIK compared to EM-SFM, CSF and RIK, respectively. It can be seen that, for different sequence sizes, SSI-RIK generally achieves a better performance than EM-SFM, CSF and RIK.

**Table 10:** The correlation coefficients, and the corresponding mean (*μ*) and standard deviation (*σ*), when the sequence sizes vary from 7 to 14 for one individual.

	EM-SFM	CSF	RIK	SSI-RIK
7	0.3403	0.3531	0.4884	0.8279
8	0.4070	0.3740	0.4807	0.7392
9	0.5498	0.0557	0.3836	0.6499
10	0.5487	0.5362	0.1682	0.6713
11	0.5513	0.7893	0.7490	0.4687
12	0.5740	0.8234	0.4467	0.6623
13	0.5978	0.8129	0.7675	0.7120
14	0.6020	0.1920	0.5513	0.6531
*μ*	0.5214	0.4921	0.5044	0.6731
*σ*	0.0952	0.2968	0.1939	0.1019

**Table 11:** The *z*-coordinate errors, and the corresponding mean (*μ*) and standard deviation (*σ*), when the sequence sizes vary from 7 to 14 for one individual.

	EM-SFM	CSF	RIK	SSI-RIK
7	0.8670	0.4939	0.1774	0.1152
8	0.9005	0.3342	0.2935	0.2265
9	1.1727	0.5746	0.6061	0.2901
10	1.1730	0.1737	0.2184	0.3266
11	1.1560	0.2191	0.1494	0.3216
12	1.1092	0.1100	0.4518	0.2804
13	1.0099	0.1507	0.1296	0.1813
14	1.0289	0.4231	0.5520	0.2551
*μ*	1.0522	0.3099	0.3223	0.2496
*σ*	0.1212	0.1730	0.1889	0.0727

**Table 12:** The correlation coefficient increasing percentages (%) of EM-SFM, CSF and RIK to SSI-RIK, when the sequence sizes vary from 7 to 14 for one individual.

	$(\frac{SSI-RIK}{EM-SFM}-1)*100$	$(\frac{SSI-RIK}{CSF}-1)*100$	$(\frac{SSI-RIK}{RIK}-1)*100$
7	143.2793	134.5014	69.5317
8	81.5967	97.6609	53.7704
9	18.1888	1066.9	69.4185
10	22.3452	25.1935	299.1271
11	-14.9772	-40.6099	-37.4219
12	15.3750	-19.5635	48.5639
13	19.0956	-12.4199	-7.2393
14	8.5008	240.2560	18.4719
*μ*	29.0906	36.7832	33.4294

**Table 13:** The *z*-coordinate error decreasing percentages (%) of EM-SFM, CSF and RIK to SSI-RIK, when the sequence sizes vary from 7 to 14 for one individual.

	$(1-\frac{SSI-RIK}{EM-SFM})*100$	$(1-\frac{SSI-RIK}{CSF})*100$	$(1-\frac{SSI-RIK}{RIK})*100$
7	84.7816	76.6792	35.0948
8	71.2805	32.2333	22.8317
9	76.8843	49.5067	52.1282
10	73.9893	-88.0403	-49.5110
11	74.0310	-46.7908	-115.2227
12	76.4741	-154.7749	37.9501
13	83.4065	-20.2712	-39.8828
14	77.0480	39.7177	53.7929
*μ*	77.0231	19.4680	22.5591

**Fig 7:**
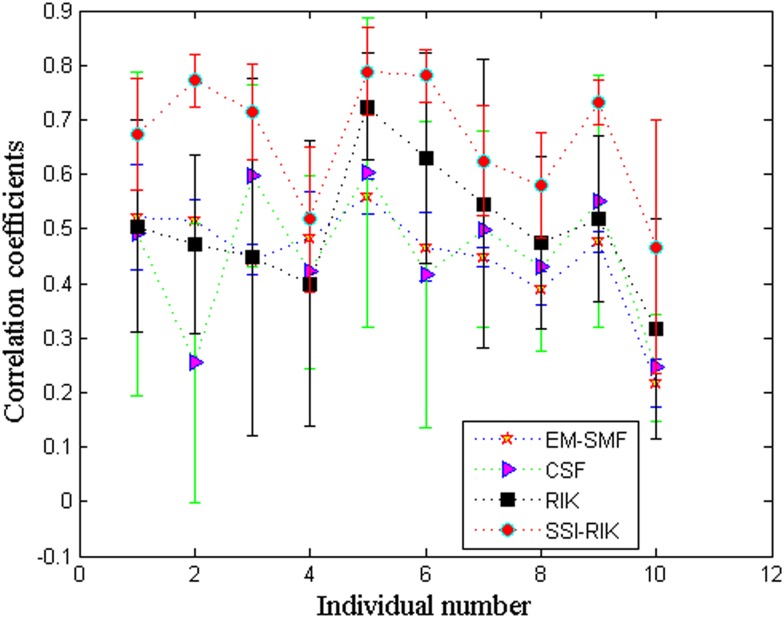
The overall mean and standard deviation (*μ* ± *σ*) of correlation coefficients for 10 individuals.

**Fig 8:**
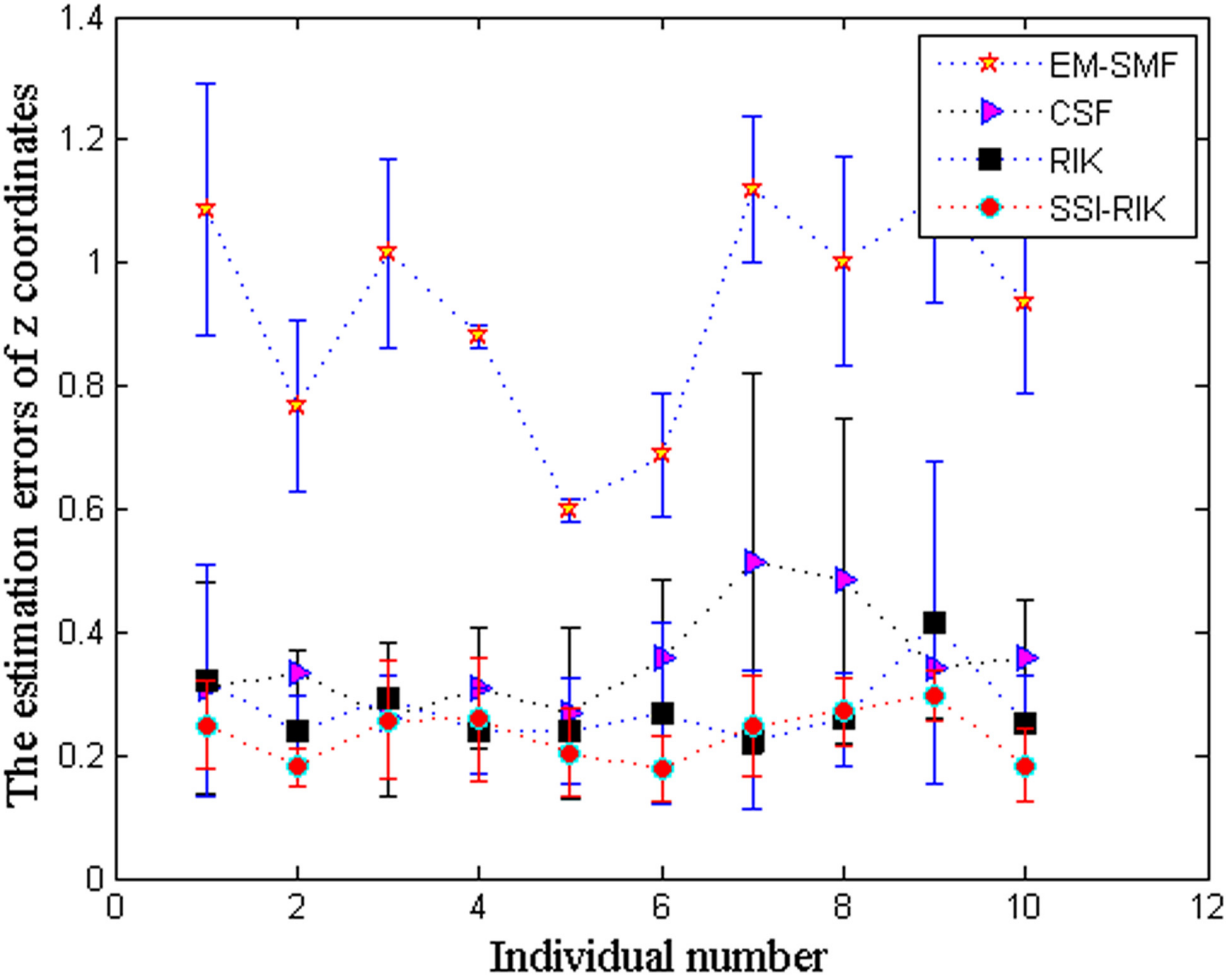
The overall mean and standard deviation (*μ* ± *σ*) of *z*-coordinate errors for 10 individuals.

Further, Figs 7 and 8 show the overall mean and standard deviation (*μ* ± *σ*) of correlation coefficients and *z*-coordinate errors for 10 individuals, respectively. In these two figures, the *x* axis denotes the individuals in terms of their corresponding number in the database. We can see that, again, SSI-RIK has a better performance than EM-SFM, CSF and RIK for different individuals.

### Discussions

There are two possible methods to integrate the outputs of the weaker estimators, i.e. the arithmetic average (denoted as AA-SSI-RIK) and the trimmed mean (denoted as TM-SSI-RIK). For the results given in Tables 10, 11 and 14 tabulates the correlation coefficients, the *z* coordinate errors, and the corresponding mean (*μ*) and standard deviation (*σ*) when the sequence sizes vary from 7to 14 using the different integration methods. Moreover, Table 15 shows the corresponding increasing and decreasing percentages of TM-SSI-RIK compared to AA-SSI-RIK. We can see that TM-SSI-RIK generally has a higher estimation accuracy than AA-SSI-RIK. Therefore, the trimmed mean is adopted in our proposed method to integrate the outputs of the weaker estimators.

**Table 14:** The correlation coefficients, *z*-coordinate errors, and the corresponding mean (*μ*) and standard deviation (*σ*), when the sequence sizes vary from 7 to 14 for different integration methods.

	correlation coefficients		*z* coordinates errors	
	AA-SSI-RIK	TM-SSI-RIK	AA-SSI-RIK	TM-SSI-RIK
7	0.6333	0.8279	0.3114	0.1152
8	0.6262	0.7392	0.4437	0.2265
9	0.6585	0.6499	0.3584	0.2901
10	0.6408	0.6713	0.4801	0.3266
11	0.5689	0.4687	0.3589	0.3216
12	0.6470	0.6623	0.3869	0.2804
13	0.6681	0.7120	0.2147	0.1813
14	0.6775	0.6531	0.2449	0.2551
*μ*	0.6400	0.6731	0.3499	0.2496
*σ*	0.0335	0.1019	0.0911	0.0727

**Table 15:** The corresponding increasing and decreasing percentages of SSI-RIK compared to EM-SFM, CSF and RIK for the results given in Table 14.

	$(\frac{AA-SSI-RIK}{TM-SSI-RIK}-1)*100$	$(1-\frac{AA-SSI-RIK}{TM-SSI-RIK})*100$
7	30.7334	63.0102
8	18.0353	48.9562
9	-1.3173	19.0484
10	4.7741	31.9808
11	-17.6120	10.3814
12	2.3555	27.5474
13	6.5665	15.5656
14	-3.5952	-4.1499
*μ*	5.1567	28.6650

As RIK has been developed originally for the long sequences, we also present here the experimental comparison of RIK and SSI-RIK when the entire sequence is used to estimate the 3D shapes. Tables 16 and 17 show the mean and standard deviation (*μ* ± *σ*) of the correlation coefficients and the *z*-coordinate errors, respectively. We can see that the performance of SSI-RIK is better than RIK for most sequences.

**Table 16:** The mean and standard deviation (*μ* ± *σ*) of the correlation coefficients when the entire sequences are used in the experiments for RIK and SSI-RIK.

	RIK	SSI-RIK
stretch	0.9421±0.0086	0.9778 ±0.0200
face1	0.9994±4.48e-4	0.9791±0.0234
face2	0.7447±0.1777	0.9209 ±0.0617
cubes	0.9793±0.0013	0.9855 ±0.0018
matrix	0.3081±0.1560	0.4577 ± 0.1912
dance	0.9065 ± 0.0132	0.9500 ± 0.0109

**Table 17:** The mean and standard deviation (*μ* ± *σ*) of the *z*-coordinate errors when the entire sequences are used in the experiments for RIK and SSI-RIK.

	RIK	SSI-RIK
stretch	0.0705±0.0103	0.0460 ±0.0232
face1	0.0079±0.0031	0.0503±0.0380
face2	0.1384±0.0539	0.0657±0.0345
cubes	0.1212±0.0118	0.0766±0.0155
matrix	0.5439±0.0724	0.2540±0.0378
dance	0.0737±0.0051	0.0622±0.0071

**Fig 9:**
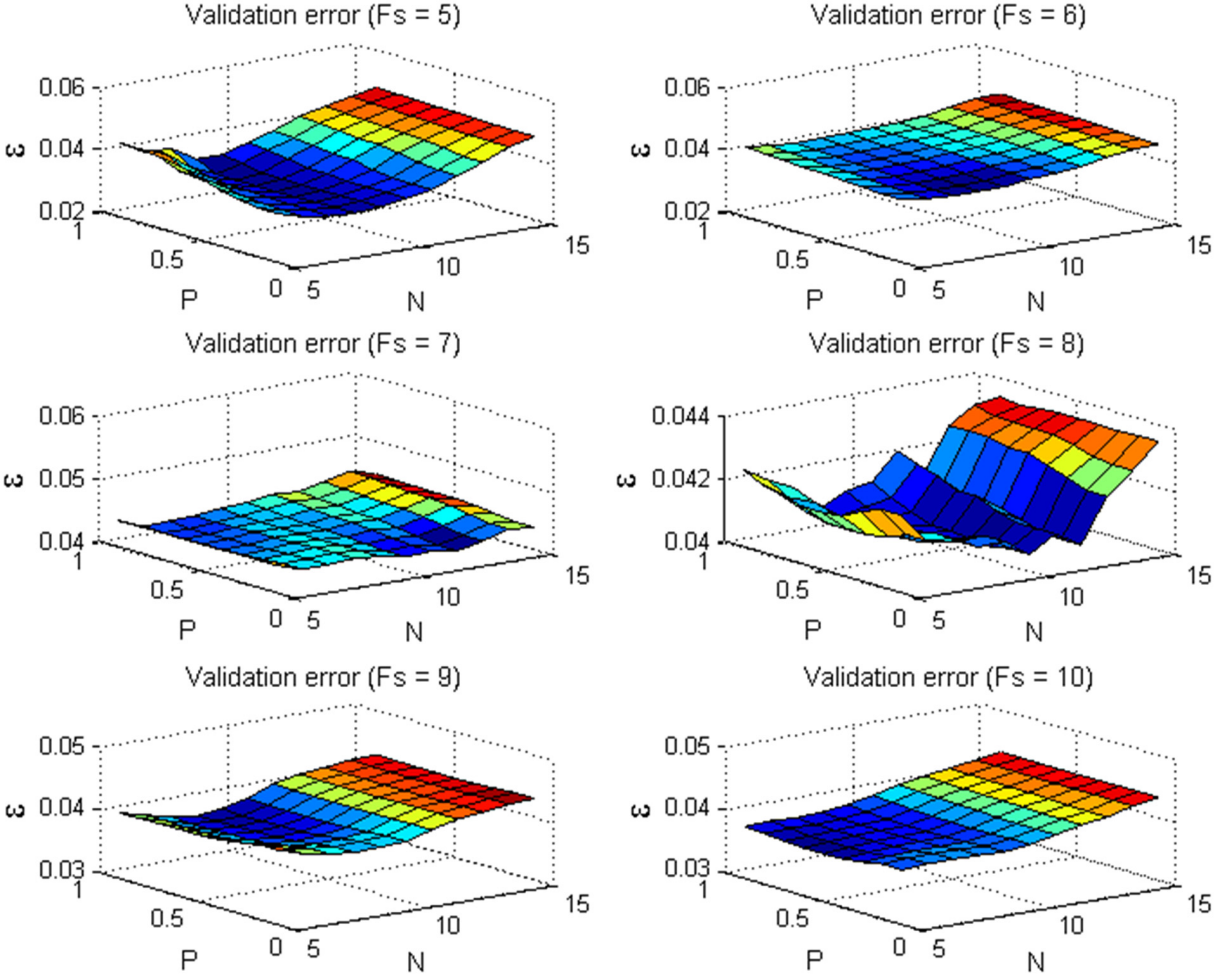
The mean *z*-coordinate errors of the 5-fold validation sets with different parameters *Fs*, *N* and *P* for the sequence *stretch*.

**Fig 10:**
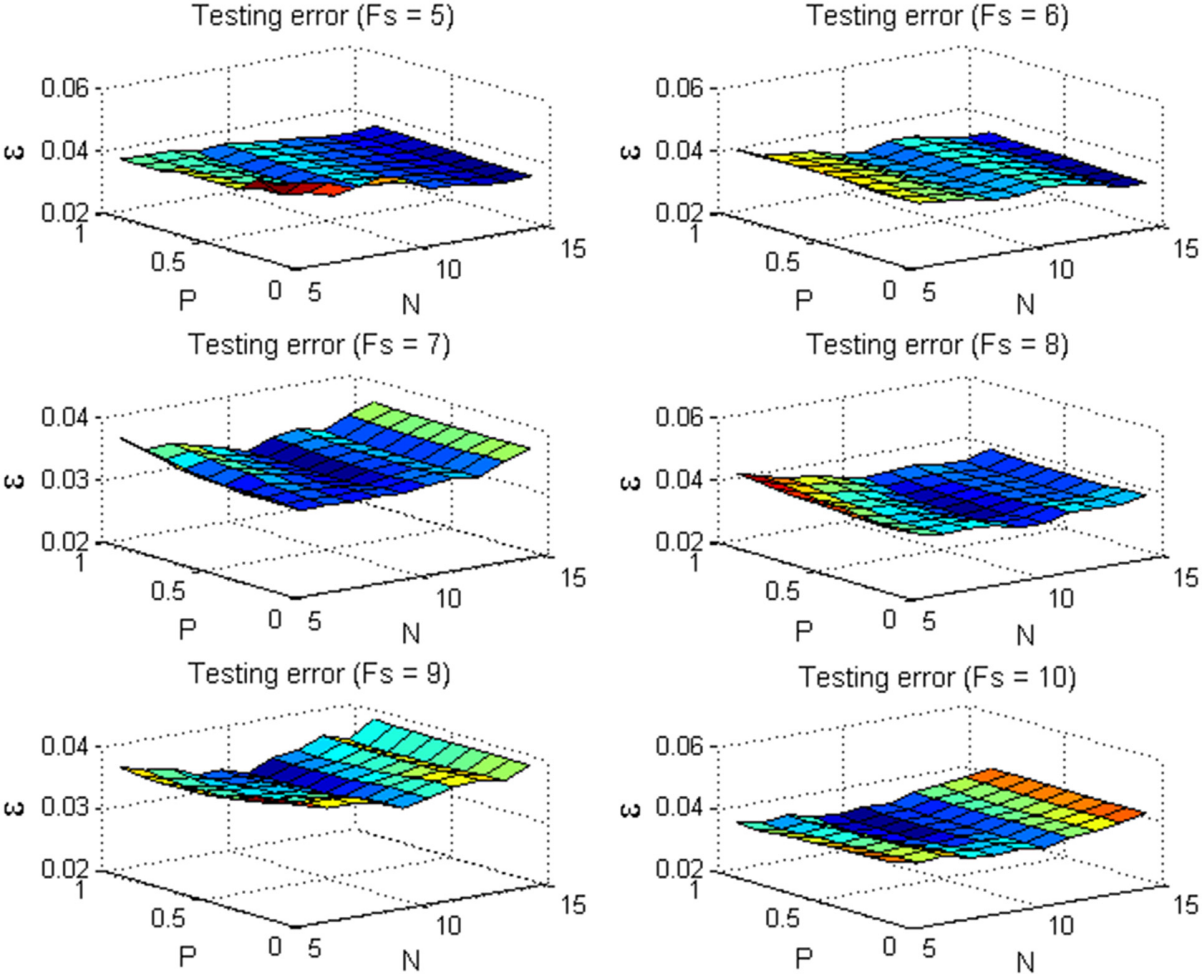
The *z*-coordinate errors of the testing sets with different parameters *Fs*, *N* and *P* for the sequence *stretch*.

Similar to pattern recognition, we tried to search for the optimal values of parameters *Fs*, *N* and *P* with the cross validation method, which is a widely used parameter selection approach. After the small-size sequences are extracted from the original sequences, the remained frames are divided into 5 folds and used as the validation sets. Furthermore, the grid divisions are carried out on the three parameters. The *z*-coordinates of the validation sets are estimated via the proposed method with each possible set of parameters *Fs*, *N* and *P*. Take the sequence *stretch* for example, Fig 9 shows the mean *z*-coordinate errors of 5-fold validation sets for different *Fs*, *N* and *P*. Correspondingly, Fig 10 shows the *z*-coordinate errors of the testing sequences. We can see that the testing error may not be small for a set of parameter with a small validation error. Thus, it is not effective to search for the optimal parameters with the cross validation method. On the other hand, it can be seen from Fig 10 that the *z*-coordinate errors vary with different parameter values, but the variations are not so significant. Besides the cross validation, there are many other parameter selection methods. Thus, how to devise a more effective method to accurately determine the optimal parameter values should be a meaningful and valuable work.

## Conclusions

In this paper, a sub-sequence-based RIK algorithm is proposed for NRSFM for small-size sequences. Compared to some existing algorithms, the proposed method has a higher estimation accuracy. Moreover, the robustness of the proposed method is better than those of the existing algorithms. The experimental results on both the artificial and the real data have verified the effectiveness and feasibility of the proposed method.
